# The endocrine adipose organ

**DOI:** 10.1007/s11154-022-09709-w

**Published:** 2022-01-19

**Authors:** Saverio Cinti

**Affiliations:** Scientific Director Center of Obesity, Marche Polytechnic University, Via Tronto 10a, 60126 Ancona, Italy

It is a real pleasure and honour for me to serve as guest editor for this special issue of REMD for several reasons.

First of all, the wide acceptance of the concept of adipose organ that is included in the title.

It is anatomy that teaches us that adipose tissues are forming the parenchyma of a real organ. There is no accepted definition for “organ”, but my forty years experience in studying and teaching anatomy to medical students convinced me that a reasonable definition could be: “any anatomical dissectible structure composed by at least two different tissues with different functional roles, but cooperating each other for a unitary finalistic role”. For example stomach is an organ dissectible and made by different tissues: muscles for peristalsis and glands for digestive juice. Peristalsis and digestive juice cooperate for digestion.

Surgical microscope-guided dissections showed the unequivocal dissecability of mice adipose organ in different physiological conditions [1]. Furthermore, macroscopic appearance (including colours) and histology analyses demonstrated its composition by white (WAT) and brown (BAT) adipose tissues. These data were presented for the first time at the 8^th^ International Congress of Obesity at Paris in 1998 together with the new concept of adipose organ [1].

Most scientists will agree that there are no questions about WAT and BAT different anatomy and functions i.e.: WAT: unilocular cells for energy storage, BAT: multilocular cells for thermogenesis, but what about their reciprocal cooperation? Several experiments suggested that white and brown adipocytes have an unexpected propriety: the ability to reversibly convert each other while in their mature phenotype and function. This implies that a mature cell is able to physiologically and reversibly reprogram its genetic organization and phenotype. Further experiments showed not only WAT-BAT transdifferentiation phenomena occur into the adipose organ [2, 3], but also the ability of white adipocytes of mammary glands to physiologically and reversibly convert into milk-producing epithelial cells during pregnancy and lactation (see Colleluori et al. contribution). All together, these data allow to think that the reciprocal cooperation consist in their ability to convert each other to reinforce their functional roles in particular situations: browning as reaction to chronic cold and whitening as reaction to chronic positive energy balance. Thus, the energy of fat is used for thermogenesis to combat undesired body temperature decline or for energy storage to combat possible future fasting problems, both pointing to guarantee animal survival. Furthermore, the ability of adipocytes to convert into milk secreting cells, also called pink adipocytes, during pregnancy and lactation will guarantee the survival of pups and therefore of the species.

The adipose organ plasticity could be used for therapeutic purposes because its browning has been proved to be healthy for humans [4] and the deep knowledge of cellular mechanisms responsible for mammary plasticity could help in understanding the fat role in breast cancer, that is one of the most frequent tumour in women [5].

The top level of scientists that accepted my invitation to contribute to this issue is of great relevance and satisfaction.

The knowledge of cellular composition of organs is an obvious premise to understand their physiology. New modern techniques such as high-resolution single cells/nucleus RNA seq, are now available and allowed to refines the composition of this organ. New cells with important regulatory functions have been recently identified in the adipose organ both in WAT and in BAT. Keeping in mind that the majority of cell types in the adipose organ are those forming the stroma-vascular fraction (SVF) the cellular heterogeneity of mature adipocytes, along with that of cells within the SVF, can be considered one of the major determinants of adipose organ function and plasticity (see Wang et al. contribution).

The endocrine nature of this organ is well established and the importance of produced hormones is outlined by the fact that their main target is the brain. Recently a new important hormone produced by white adipocytes has been discovered: asprosin [6, 7]. This hormone contributes together with the well-known leptin to the most important survival behaviour for all mammals: the one able to guarantee the nutritional supply. Strikingly, hormones produced by the adipose organ are responsible for this behaviour. Nevertheless leptin has several peripheral regulatory actions in the adipose organ (see Picó et al. contribution). Also the BAT component of the organ is able to produce hormones and peptide factors called batokines (fibroblast growth factor-21, neuregulin-4, phospholipid transfer protein, interleukin-6, adiponectin, myostatin), lipokines and miRNAs. Some of them influence the activity of digestive organs reinforcing the concept of inter-organs cooperation as a real nutritional system [8]. Furthermore their activity on heart and skeletal muscles underline the BAT activity for a healthy metabolism in the human system (see Gavaldà-Navarro et al. contribution). Importantly, the adipose organ has complex anatomo-functional relationships with these organs (heart and skeletal muscles) and vasculature. These anatomical aspects, including the specific cellular phenotype interaction and perfusion, must be taken into account in order to understand the adipose organ role in one of the most important metabolic consequences due to an excess of WAT accumulation: insulin resistance (see Camastra and Ferrannini contribution).

All molecular components to produce and degrade the components of the paracrine endocannabinoid system are represented in the adipose organ. Here they form a signalling complex able to regulate critical homeostatic processes, including adipogenesis, lipogenesis, lipolysis and thermogenesis. This system is a critical contributor to the hypothalamic control of adipose organ physiology. Importantly, excess of endocannabinoids activity and CB1 receptors in visceral fat has been found in obesity (see Joung et al. contribution).

Endocrine organs usually act trough secretion of hormones contained into specific organelles called granules. The adipose organ developed a mode of signalling that is even more efficient: extracellular vesicles (EV). This heterogeneous population of membrane-enclosed nano-particles containing signalling proteins, lipids, miRNAs are able to induce coordinated signalling pathways in recipient cells. Thus, EV-mediated signalling can integrate the functional activation or suppression of multiple pathways simultaneously at all levels of regulation: mRNA, protein, and post-translational modifications (see Crewe and Scherer contribution).

The pathology of adipose organ is extremely frequent. According to WHO about 650 millions of adults are obese and have a diseased adipose organ [9]. The health consequences are really serious and mainly related to type 2 diabetes (T2D) and atherosclerosis development [10]. This aspect requires maximal alert and urgent therapeutic solutions also in view to the fact that actual therapeutic approaches have poor long-term efficiency [11].

The two main alterations of obese adipose organ are: the chronic low grade inflammation [12, 13] and fibrosis [14] causing various aspect of organ dysfunction also known as adiposopathy. This concept must be extended to the all fat depots segregated in anatomical sites not contiguous with the dissectible fat depots of the adipose organ, such as into muscles, bones, thymus, hearth (see Favaretto et al. contribution). The adiposopathy could be related to the activity of cells included in the heterogeneous pool of adipose cell precursors. Thus, the studies related to the origin of adipose precursors and mechanisms influencing the fate of mesenchymal stem cells in the adipose organ is important not only for its general biological significance but also in relationship to its involvement in the pathology of this organ (see Marcelin and Clement contribution).

Interactions between adipocytes and cancer cells are another important topic and further studies are necessary to understand the precise role of EVs in this aspect (see Crewe and Scherer and Favaretto et al. contributions).

Some possible strategies to understand mechanisms useful for future therapies or direct possible therapeutic approaches are suggested in this issue. Together with the well known healthy properties of browning of adipose organ [4] in this fascicle also new targets and new strategies have been reviewed.

Leptin-resistance must be obviously tackled and we need to understand the pathways used by leptin to exert its anorectic potentials. CNTF, enhancing the capacity of the hormone to get access into the brain, can help in this direction. Furthermore, an improvement of leptin sensitivity can be obtained enhancing the capacity of brain circuits to undergo neuronal plasticity (see Maffei and Giordano contribution).

Blocking the activity of endocannabinoids CB1 receptors targeting adipose and other peripheral organs is another focus of preclinical and clinical research that deserves further studies (see Jung et al. contribution).

Another fascinating possible strategies include the brown fat transplantation. Creation of brown adipospheres for cell based transplant therapy is now technically possible and several steps in this direction have already been performed, thus increasing the possibilities of therapeutic perspectives (see Dani et al. contribution).

Another interesting new avenue for therapeutic horizons come from the unexpected discovery that the adipose organ express taste and light receptors. These receptors own to the super family of G-coupled receptors and are present in both WAT and BAT components. They seem to be able to influence different aspects of adipose organ biology including browning of fat cells (see Ekechukwu and Christian contribution).

The basal metabolic rate can be regulated to reduce energy dissipation during periods of decreased nutrient intake. This implies the need for compensatory mechanisms to fine-tune the amount of energy required to sustain the metabolic processes. Understanding the regulation of energy-dissipating processes modulated by food intake, including the UCP1-independent mechanisms of mitochondrial uncoupling and ATP-consuming futile cycles, can potentially lead to therapeutic strategies to promote weight loss among individuals with obesity (See Brownstein et al. contribution).

Furthermore, the reported association of T2D incidence with the abundance of adipose progenitor cells (denominated P1 and P4) in subcutaneous and visceral depots, respectively, is of high interest, as these cell types are characterized by lower gene expression related to PPARγ, which has been reported to play a protective role in the development of insulin resistance. Thus, targeting these cell subtypes could be a novel strategy to treat T2D (see Wang et al. contribution).

**Fig. 1:**
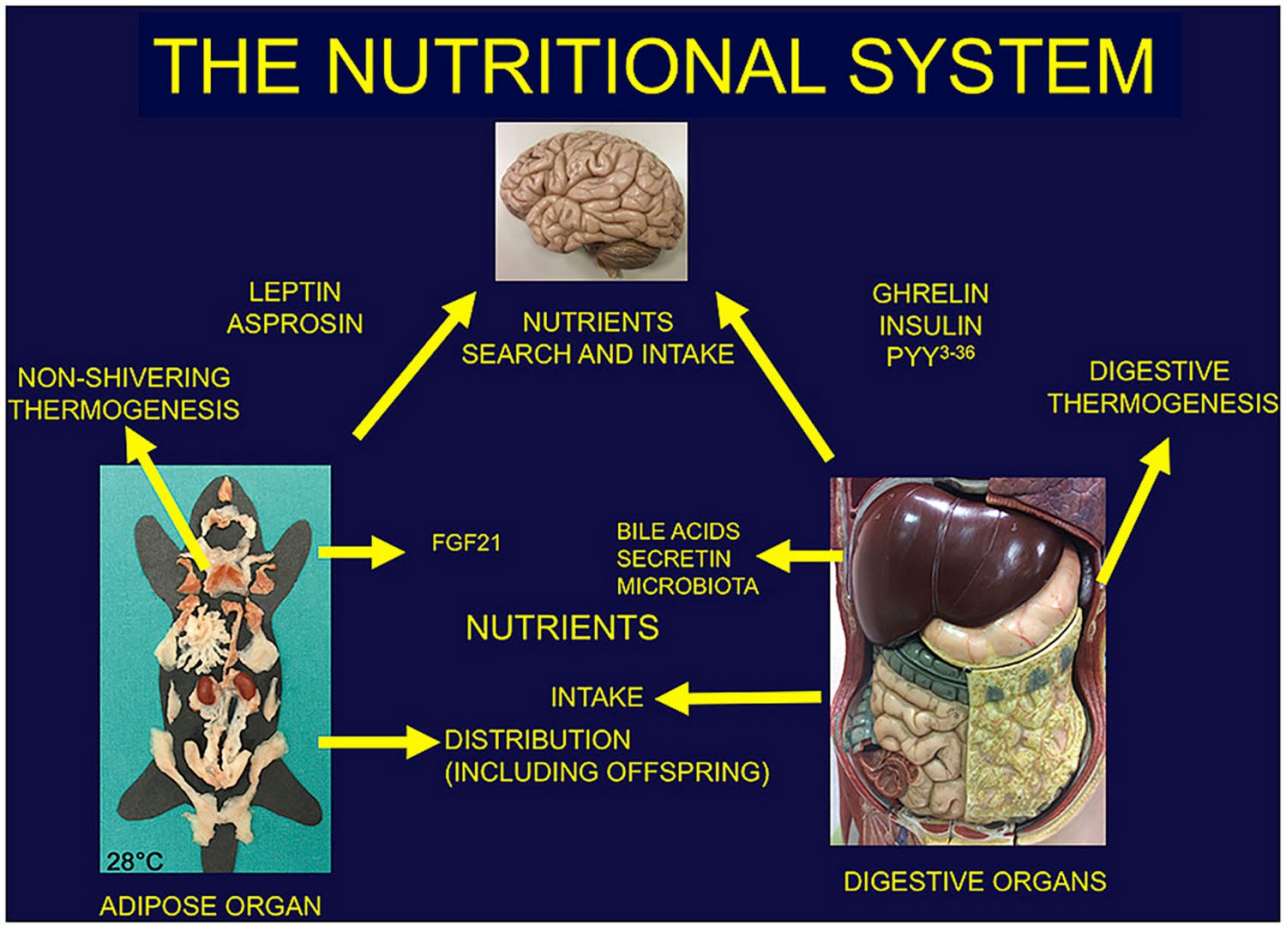
The nutritional system concept (from Ref 8 with permission)

In conclusion, we can safely say that mammals have a new organ: the adipose organ. Usually, organs are organized into systems (nervous, cardiovascular, digestive, urinary, reproductive, etc.), able to perform complex functional activities. Recently, I proposed a new concept: the nutritional system [8]. The adipose organ and digestive organs form this system (Fig. 1). They both influence brain to search and intake food by hormones. They produce factors reciprocally influencing thermogenesis (also important to modulate food intake, especially in new-borns [15]). Finally, they collaborate for the intake and distribution of nutrients, including distribution of milk to pups (see Colleluori et al. contribution), thus allowing not only survival of individuals (short term homeostasis) but also of the species (long term homeostasis).

Lastly, I take this opportunity to thank you all Authors that dedicated their knowledge and time to provide their updated reviews included in this issues. Their names are a guarantee at international top-level, thus conferring to this issue a very high quality message for the readers. I also thank you very much all Authors that were willing to give their contributions but, mainly due to the contingent COVID-19 pandemic situation, at the end declined.

A special thanks is due to Georgia Colleluori that helped me a lot in this editorial job and Felipe Casanueva for his invitation to be Guest Editor. I am linked to Prof Casanueva by a special friendship born in several meeting occasions that raised a deep awareness of growing mutual scientific and human esteem.
